# Rapid development of double-hit mRNA antibody cocktail against orthopoxviruses

**DOI:** 10.1038/s41392-024-01766-8

**Published:** 2024-03-27

**Authors:** Hang Chi, Suo-Qun Zhao, Ru-Yi Chen, Xing-Xing Suo, Rong-Rong Zhang, Wen-Hui Yang, Dong-Sheng Zhou, Min Fang, Bo Ying, Yong-Qiang Deng, Cheng-Feng Qin

**Affiliations:** 1 State Key Laboratory of Pathogen and Biosecurity, Beijing Institute of Microbiology and Epidemiology, AMMS, 100071 Beijing, China; 2 College of Life Sciences, Inner Mongolia Agricultural University, Hohhot, 010018 Inner Mongolia China; 3 School of Life Sciences, Henan University, Kaifeng, 475004 Henan China; 4 Suzhou Abogen Biosciences Co., Ltd, Suzhou, 215123 Jiangsu China; 5 Research Unit of Discovery and Tracing of Natural Focus Diseases, Chinese Academy of Medical Sciences, 100071 Beijing, China

**Keywords:** Nucleic-acid therapeutics, Microbiology

## Abstract

The *Orthopoxvirus* genus, especially variola virus (VARV), monkeypox virus (MPXV), remains a significant public health threat worldwide. The development of therapeutic antibodies against orthopoxviruses is largely hampered by the high cost of antibody engineering and manufacturing processes. mRNA-encoded antibodies have emerged as a powerful and universal platform for rapid antibody production. Herein, by using the established lipid nanoparticle (LNP)-encapsulated mRNA platform, we constructed four mRNA combinations that encode monoclonal antibodies with broad neutralization activities against orthopoxviruses. In vivo characterization demonstrated that a single intravenous injection of each LNP-encapsulated mRNA antibody in mice resulted in the rapid production of neutralizing antibodies. More importantly, mRNA antibody treatments showed significant protection from weight loss and mortality in the vaccinia virus (VACV) lethal challenge mouse model, and a unique mRNA antibody cocktail, Mix2a, exhibited superior in vivo protection by targeting both intracellular mature virus (IMV)-form and extracellular enveloped virus (EEV)-form viruses. In summary, our results demonstrate the proof-of-concept production of orthopoxvirus antibodies via the LNP-mRNA platform, highlighting the great potential of tailored mRNA antibody combinations as a universal strategy to combat orthopoxvirus as well as other emerging viruses.

## Introduction

The genus *Orthopoxvirus* contains a number of species that can cause significant pox diseases in humans and animals, including the monkeypox (mpox)-causing agent monkeypox virus (MPXV), the cowpox-causing agent cowpox virus (CPXV), and the most well-known smallpox-causing agent variola virus (VARV).^1^ VARV killed an estimated 300–500 million people (mortality rate, ~30%) in the 20th century. After a historic global vaccination campaign, smallpox was officially declared eradicated worldwide by 1980.^2^ Today, *Orthopoxvirus* infections remain a significant public health threat.^3–5^ Since May 2022, mpox has suddenly spread to many countries without documented mpox transmission, and new cases have been continuously reported over time, giving rise to global concern regarding the current mpox outbreak.^6,7^ As of 31 November 2023, a cumulative total of 92,783 laboratory-confirmed cases of mpox, including 171 deaths, from 116 countries and regions, have been reported to the WHO (https://www.who.int/health-topics/monkeypox/). Today, the unprecedented multicountry mpox outbreak is still ongoing. As a DNA virus, MPXV theoretically will not frequently mutate like RNA viruses. However, since the adaptive mutations have been found in the genomes of recent mpox outbreaks, the possibility of MPXV further adaptive mutations cannot be ruled out in the context of the worldwide monkeypox outbreak, which may lead to a larger-scale outbreak.^8,9^ Besides, if the MPXV of Central African Clade is spread which is more severe, it may result in far more devastating outcomes. Furthermore, the smallpox-causing agent VARV considered a major threat to humans due to its possible use as a bioterrorism agent and the potential possibility of future re-emergence.^10,11^ Thus, there is an urgent need for developing effective therapeutic strategies to combat orthopoxviruses.

Orthopoxviruses are complex dsDNA viruses with over 200 genes encoding many proteins, including enzymes and factors necessary for self-replication and maturation. Intracellular mature virus (IMV) and extracellular enveloped virus (EEV) are two main antigenically distinct infectious enveloped virions that exist during infection. The former is released from infected cells through the classical exocytosis pathway in the early stages of infection, while the latter is released by lysis in the late stages.^12^ Several EEV and IMV surface proteins play crucial roles in viral cycle and eliciting immune response. The VACV A33, B5 proteins are involved in EEV formation and subsequent transmission, whereas the MPXV M1 protein and VACV A29 protein are involved in cellular entry of the mature virion.^13^ Besides, viral antigens in the virus envelope, such as B5, A33, A27, L1, D8, and H3, are crucial for eliciting protective antibodies. However, although orthopoxviruses induce cross-reactive antibodies that protect against infection from other orthopoxvirus species, the discontinued smallpox vaccination leaves a large part of the world population with no immunity against smallpox or other zoonotic orthopoxvirus infections, which highlights the need for effective treatments to control the current and future outbreaks of human poxviruses.^14^

To date, various therapeutics, including antiviral inhibitors, human vaccinia immune globulin (VIG) and poxvirus-specific monoclonal antibodies, have been developed and tested for their efficiency against poxvirus infection. Two small-molecule inhibitors, tecovirimat (also known as ST-246 or TPOXX) and brincidofovir (Tembexa), were approved by the United States (US) Food and Drug Administration (FDA), in July 2018 and June 2021, respectively, for the treatment of human smallpox. In addition, two intravenous formulations of VIG, VIGIV Cangene and VIGIV Dynport, were licensed for the management of human VACV infection and complications associated with the smallpox vaccine.^15,16^ Among these therapeutics, antibody therapies for orthopoxvirus remarkably improved survival by reducing weight loss and tissue viral burden and successfully protected mice against severe disease.^17–19^ Notably, there is growing evidence of the importance of poxvirus antibodies in virus control and recovery from primary and secondary infections.^20^ The development of therapeutic monoclonal antibodies represents a promising strategy to prevent or treat orthopoxvirus infection. To date, several protective monoclonal antibodies (mAbs) targeting EEV and IMV surface proteins have been identified and exhibited potent antiviral efficacy in vitro and in animal models.^21–24^ mAbs 22, 283, 26, and 301, respectively targeting VACV A33, VACV B5, MPXV M1, and VACV A27 protein, were identified from a panel of orthopoxvirus-specific human mAbs derived from donors recovered from MPXV infection or immunized with one of three different vaccines. These four original protein format antibodies exhibited broadly cross-neutralizing activity against several orthopoxvirus species tested, including VACV, CPXV, MPXV, and VARV.^25^ However, none of these antibodies have been approved for clinical use.

Recently, mRNA-encoded antibody for prophylactic or therapeutic purpose against infectious diseases has become an attractive approach. As an alternative avenue to passive immunotherapy, mRNA-antibody approach presents a range of unique advantages such as simple and cost-effective production, immediate protection, as well as flexibility, making it well-suited for rapid response to emerging pandemics.^26,27^ Herein, we report the first mRNA antibody against orthopoxvirus with in vivo protection. By using a recently established lipid nanoparticle (LNP)-encapsulated mRNA platform, we constructed a panel of mRNAs that encode broad neutralization antibodies against orthopoxvirus (mAbs 22, 283, 26, and 301). A single intravenous injection of each mRNA antibody rapidly produced potent neutralizing antibodies in mice. More importantly, a double-hit mRNA antibody cocktail, Mix2a, which targeted both the EEV-form and IMV-form viruses, showed superior protection potency against lethal VACV challenge in mice.

## Results

### Construction and characterization of orthopoxvirus antibody-encoding mRNAs

**Fig. 1:**
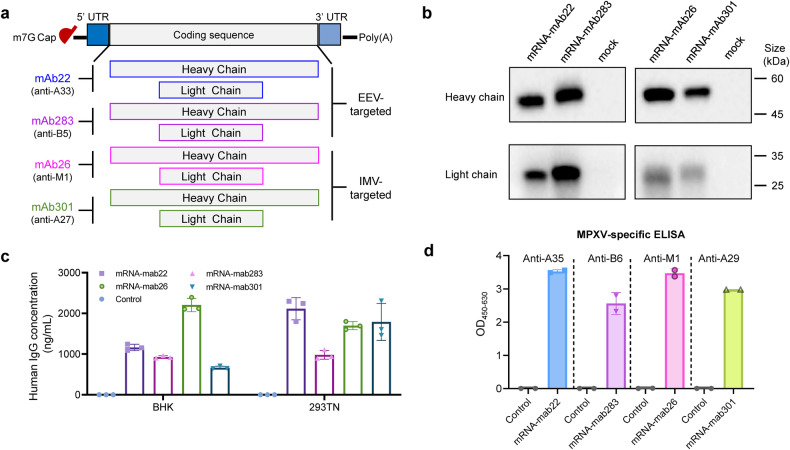
Construction and characterization of anti-orthopoxvirus monoclonal antibody-encoding mRNAs. **a** Schematic of mRNAs encoding the heavy and light chains of the anti-orthopoxvirus monoclonal antibody (mAb). IgK Sp, human IgK light chain signal peptide. mAb22, mAb283, mAb301 and mAb26 target VACV A33, B5, MPXV M1 and VACV A27 proteins, respectively. **b** Validation of mAb heavy and light chain expression in the corresponding mRNA-transfected cell supernatants by Western blot. BHK-21 cells were transfected with HC mRNA and LC mRNA. The supernatants were harvested at 24 h post transfection. **c** Quantification of mAb levels in mRNA transfection supernatants using human IgG ELISA. BHK-21 cells and 293TN cells were respectively transfected with HC mRNA and LC mRNA. Transfection supernatants were harvested at 24 h post transfection. **d** The evaluation of binding activity respectively to MPXV proteins A35, B6, M1, and A29 of the BHK-21 cell transfection supernatants using indirect ELISA. The absorbance at 450 nm /630 nm was measured. Data are shown as the mean ± SD. Data were obtained using GraphPad Prism version 9.0 (GraphPad software)

The members of the *Orthopoxvirus* genus have been proven to be antigenically closely related.^28^ To develop panorthopoxvirus mRNA antibodies, we first chose four cross-neutralizing mAbs, mAb22, mAb283, mAb26, and mAb301, that target VACV-A33, VACV-B5, MPXV-M1 and VACV-A27 protein, respectively. These four original protein format antibodies were proven to be cross-protective against MPXV, VACV, VARV and CPXV. The homology of the four targeted antigens in our study among these four viruses is shown in Supplementary Table 1. The coding sequences of both the heavy chain (HC) and light chain (LC) of each candidate mAb were optimized and inserted into the plasmid ABOP-028, respectively. The final four mRNA combinations were prepared as previously described.^29^ Utilizing the well-established mRNA platform, mRNA constructs containing the 5’ ^7m^G^PPP^G cap structure, 5’ untranslated region (UTR), signal peptide, codon optimized coding sequence of candidate mAbs, 3’ UTR, and poly(A) tail were prepared (Fig. 1a). The in vitro expression of the mRNA combination of each candidate mAb was first confirmed in BHK-21 cells, and the Western blotting results showed that both HC and LC proteins were readily detected as expected 24 h post transfection (Fig. 1b). Additionally, the IgG antibody concentration in the supernatants from different mammalian cell lines (BHK-21 and 293TN) transfected with the corresponding mRNA combinations ranged from 665 to 2206 ng/ml, as determined by human IgG ELISA (Fig. 1c). To identify the antigen-binding activities of the supernatants, MPXV EEV and IMV surface proteins were selected as representative antigens. Further ELISA detection showed that the mAb produced from mRNA combinations were respectively capable of binding to A35, B6, M1, and A29 of MPXV (Fig. 1d). These data demonstrate that the mRNA combinations are functional in producing mRNA-launched antibodies.

### Characterization of the LNP-encapsulated mRNAs in vivo

**Fig. 2:**
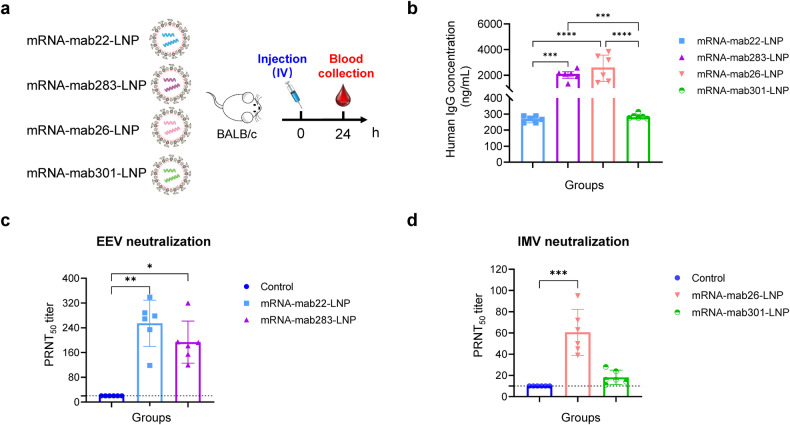
In vivo expression of LNP-encapsulated mRNA encoding protective antibodies against Orthopoxvirus. **a** Schematic of the experimental design. Six- to eight-week-old specific pathogen-free female BALB/c mice (*n* = 6 per group) were intravenously (i.v.) injected with a single dose of 1 mg/kg of mRNA-mab22-LNP, mRNA-mab283-LNP, mRNA-mab26-LNP, mRNA-mab301-LNP or placebo. Orbital blood from each group of mice were collected at 24 h post injection. Sera were harvested and heat inactivated before further test. **b** Quantification of mAb levels in sera were performed using human IgG ELISA. Statistical significance was analyzed by ordinary one-way ANOVA (***P < 0.001 and ****P < 0.0001). **c** EEV neutralization activity of mRNA-mab22-LNP and mRNA-mab283-LNP were measured using EEV forms of VACV by PRNT assay in BS-C-1 cells. **d** IMV neutralization activity of mRNA-mab26-LNP and mRNA-mab301-LNP were measured using IMV forms of VACV by PRNT assay in BS-C-1 cells. Data are shown as the mean ± SD. Statistical significance was analyzed by Kruskal‒Wallis one-way ANOVA (*p < 0.05, **p < 0.01 and ***p < 0.001). Data were obtained using GraphPad Prism version 9.0 (GraphPad software)

The four mAb-encoding mRNA combinations were further processed into the final LNP formulation as previously described.^29^ The resulting mRNA antibodies were termed mRNA-mab22-LNP, mRNA-mab283-LNP, mRNA-mab26-LNP, and mRNA-mab301-LNP in our study, and dynamic light-scattering analyses showed uniform particle sizes ranging from 73.12 to 75.39 nm with a polydispersity index (PDI) below 0.1 (Supplementary Fig. 1). To investigate the in vivo expression pattern, the four mRNA-LNP formulations were intravenously injected to BALB/c mice at a single dose. Empty LNPs were used as a placebo. Sera from each group of mice were collected at 24 h post injection (Fig. 2a). Human IgG concentrations in the mouse sera were assessed using ELISA. As shown in Fig. 2b, the serum human IgG exhibited distinct concentrations ranging from 269 to 2598 ng/mL.

To further determine whether these mRNA antibodies were fully active, plaque reduction neutralization tests (PRNTs) were performed with VACV EEV and IMV in BS-C-1 cells. As expected, high levels of anti-EEV neutralizing antibodies were measured in the sera of mRNA-mab22-LNP and mRNA-mab283-LNP injected mice (Fig. 2c), while mRNA-mab26-LNP and mRNA-mab301-LNP injection contributed to the neutralization of the IMV form of VACV (Fig. 2d). Given the neutralization titer of EEV candidates (mRNA-mab22-LNP and mRNA-mab283-LNP) is much higher than IMV candidates (mRNA-mab26-LNP and mRNA-mab301-LNP) in our study, we speculated EEV candidates is crucial for superior protection efficacy. Besides, as mRNA-mab26-LNP injection yields higher levels of IMV neutralizing mAb in vivo than mRNA-mab301-LNP, mRNA-mab26-LNP was selected as the IMV antibody component. Thus, both representatives of EEV (mRNA-mab22-LNP and mRNA-mab283-LNP) were used in combination with mRNA-mab26-LNP in the further rational design of the double-hit mRNA antibody cocktail.

### Protection efficiency of the candidate mRNA antibody component against VACV infection in the mouse model

**Fig. 3:**
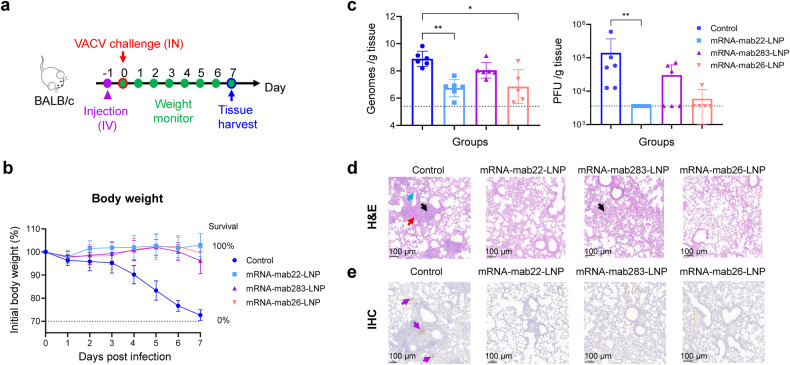
Protection efficiency of the candidate mRNA-encoded antibody component in a VACV challenge mouse model. **a** Schematic of the experimental design. Six- to eight-week-old specific pathogen-free female BALB/c mice (*n* = 5-6 per group) were intravenously (i.v.) administered a single dose of mRNA-mab22-LNP, mRNA-mab283-LNP, mRNA-mab26-LNP or placebo. All mice were intranasally challenged with 7.5 × 10^4^ PFU VACV (WR strain) at 24 h post administration and monitored daily for body weight. Tissues were harvested at 7 d.p.i. Part of lung tissues were homogenized using a tissue homogenizer. The other part of lung tissues from mice were fixed with 4% formaldehyde and embedded in paraffin. Sections of the paraffin-embedded tissues were cut and placed on glass histology slides. **b** Body weight and survival were monitored daily for 7 d.p.i. **c** Viral genome copies and VACV titers in the lung at 7 d.p.i. were measured by qPCR and standard plaque assay in BS-C-1 cells, respectively. Viral genome copies were expressed as genomes/g tissue. Virus titers were expressed as PFU/g of tissue. Data are shown as the mean ± SD. Statistical significance was analyzed by Kruskal‒Wallis one-way ANOVA (*p < 0.05 and **p < 0.01). Data were obtained using GraphPad Prism version 9.0 (GraphPad software). **d** H&E staining of the lung sections from mice infected with VACV (WR strain) at 7 d.p.i. Diffuse degeneration and necrosis of the epithelial lining (black arrow), accompanied by hemorrhage, edema (red arrow), and fibrin exudation into surrounding alveoli (blue arrow). **e** Immunohistochemistry (IHC) analysis was performed with a human anti-VACV D8L monoclonal antibody. Purple arrows indicate VACV infection foci. The slides were scanned with a Pannoramic MIDI histoscanner (3DHISTECH), and images were analyzed using Pannoramic Viewer software. Scale bars (100 μm) are indicated for each picture. Brown-colored staining indicates positive results

To assess the protective capacity of the candidate mRNA antibody component against orthopoxvirus infection in vivo, the VACV Western Reserve (WR) strain lethal challenge mouse model was used in the current study. BALB/c mice were injected intravenously with mRNA-mab22-LNP, mRNA-mab283-LNP, mRNA-mab26-LNP, or placebo one day before lethal intranasal challenge with 7.5 ×10^4^ PFU VACV. Body weight and survival were recorded daily for 7 days post challenge. Mice succumbed at 7 days post infection (d.p.i.), and tissues were harvested (Fig. 3a). As shown in Fig. 3b, mRNA antibody treatments provided complete protection against weight loss and mortality throughout the observation period, while in contrast, the body weight of mice from the control group experienced severe illness and exhibited a significant weight loss of approximately 30% of initial weight at 7 d.p.i. Moreover, high levels of viral DNA genome (~10^8.9^, ~10^8.7^, and ~10^5.7^ genomes/g tissue, respectively) were measured in lungs, nasal turbinates and livers from mice in the control group at 7 d.p.i. In contrast, mice that received mRNA antibodies showed a lower average viral DNA genome compared with that of the control group (Fig. 3c and Supplementary Fig. 2a). As lung tissue is the main infected organ in mice infected with VACV, the infectious virus particles in the lungs of infected mice were further determined using a standard plaque assay in BS-C-1 cells. As shown in Fig. 3c, treatment with mRNA-mab22-LNP resulted in complete control of lung VACV replication, with no detectable infectious virus particles. Moreover, the pathological lung lesions were investigated using H&E staining and immunohistochemistry (IHC) analysis. H&E staining results showed that VACV infection led to mild to marked diffuse degeneration and necrosis of the epithelial lining (black arrow), accompanied by hemorrhage, edema (red arrow), and fibrin exudation into surrounding alveoli (blue arrow) in the control mice, while mRNA-encoded antibody treatment, except for mRNA-mab283-LNP, significantly prevented VACV-induced lung damage (Fig. 3d). Furthermore, IHC analysis performed using anti-VACV D8 mAb showed D8L protein-positive cells in the lungs of mice from the control group, whereas no viral protein-positive cells were detected in the lung tissues of the mRNA antibody treatment group (Fig. 3e).

### Double-hit mRNA antibody cocktail contributes to functionally neutralizing antibodies and superior protection potency against *Orthopoxvirus* in mice

**Fig. 4:**
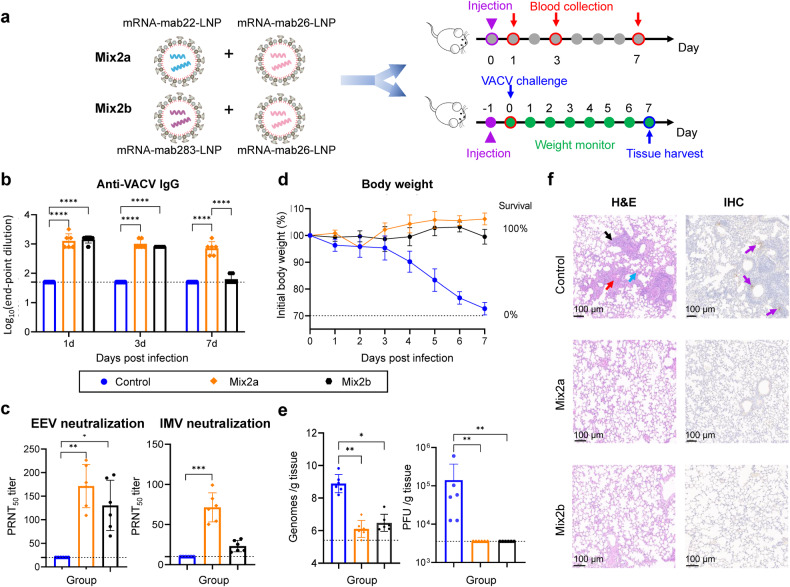
Injection of BALB/c mice with mRNA antibody cocktail contributes to neutralizing activity against Orthopoxvirus. **a** Schematic of the experimental design for the characterization of the mRNA antibody cocktail in two batches of animals. Six- to eight-week-old specific pathogen-free female BALB/c mice (*n* = 6 per group) were intravenously (i.v.) administered 2 mg/kg Mix2a (1 mg/kg mRNA-mab22-LNP plus 1 mg/kg mRNA-mab26-LNP), Mix2b (1 mg/kg mRNA-mab22-LNP plus 1 mg/kg mRNA-mab301-LNP) or placebo. In batch I, orbital blood from each group of mice were collected on Days 1, 3, and 7 post injection. Sera were harvested and heat inactivated before further test. In batch II, mice were intranasally challenged with 7.5 × 10^4^ PFU VACV (WR strain) at 24 h post administration and monitored daily for body weight. Tissues were harvested at 7 d.p.i. Part of lung tissues were homogenized using a tissue homogenizer. The other part of lung tissues from mice were fixed with 4% formaldehyde and embedded in paraffin. Sections of the paraffin-embedded tissues were cut and placed on glass histology slides. **b** The anti-VACV IgG antibody titers of sera were evaluated by indirect ELISA at the indicated times. **c** Neutralizing activity of sera at 24 h post administration was assessed using VACV IMV- and EEV-neutralization assays in BS-C-1 cells. **d** Body weight and survival were monitored daily for 7 days. **e** Viral genome copies in the lung were measured by qPCR and are expressed as genomes/g tissue. VACV titers in the lung were assessed using a standard plaque assay in BS-C-1 cells. Viral genome copies were expressed as genomes/g tissue. Virus titers were expressed as PFU/g of tissue. Data are shown as the mean ± SD. Statistical significance was analyzed by Kruskal‒Wallis one-way ANOVA (*p < 0.05, **p < 0.01, ***p < 0.001 and ****p < 0.0001). Data were obtained using GraphPad Prism version 9.0 (GraphPad software). **f** H&E staining of the lung sections from mice infected with VACV (WR strain) at 7 d.p.i. Diffuse degeneration and necrosis of the epithelial lining (black arrow), accompanied by hemorrhage, edema (red arrow), and fibrin exudation into surrounding alveoli (blue arrow). IHC analysis was performed with a human anti-VACV D8L monoclonal antibody. Purple arrows indicate VACV infection foci. The slides were scanned with a Pannoramic MIDI histoscanner (3DHISTECH), and images were analyzed using Pannoramic Viewer software. Scale bars (100 μm) are indicated for each picture. Brown-colored staining indicates positive results

To determine the best double-hit mRNA antibody cocktail to neutralize both IMV-form and EEV-form viruses, two candidates termed Mix2a (mRNA-mab22-LNP plus mRNA-mab26-LNP) and Mix2b (mRNA-mab283-LNP and mRNA-mab26-LNP) were prepared accordingly. Two batches of BALB/c mice were used for the evaluation of the Mix2a and Mix2b. Batch I was used for the characterization of the mRNA antibody cocktail in vivo, and batch II were used for the evaluation of protective efficiency against VACV challenge (Fig. 4a). In batch I, sera were collected on days 1, 3, and 7 post injection. The anti-VACV IgG antibody titers of sera were evaluated using ELISA. The mean peak concentrations of sera from the Mix2a and Mix2b groups both occurred on day 1 post injection (Fig. 4b). As shown in Fig. 4c, Mix2a and Mix2b contributed to functionally neutralizing antibodies against both the EEV form and the IMV form of VACV. Notably, Mix2a produced higher functional antibodies than Mix2b. In batch II, mice were intranasally challenged with 7.5 × 10^4^ PFU VACV WR strain and monitored daily for body weight changes throughout a 7-day follow-up. As expected, both Mix2a and Mix2b injections protected mice from losing weight after infection with a lethal dose of VACV. Apparently, mice that received Mix2a showed increased body weight gain, while mice from the Mix2b group demonstrated a relatively stable body weight (Fig. 4d). Moreover, Mix2a and Mix2b exhibited protective potency in the clearance of infectious virus particles in lung tissues (Fig. 4e) and completely prevented VACV-induced pathological lung lesions, with no viral protein-positive cells detected via IHC analysis (Fig. 4f). In addition, Mix2a demonstrated superior efficacy over Mix2b in viral DNA genome clearance (Fig. 4e and Supplementary Fig. 2b). Overall, these results suggest that optimal protection against orthopoxvirus infection requires the cooperation of both EEV- and IMV-targeted neutralizing antibodies, and highlight Mix2a as the most efficient double-hit mRNA antibody cocktail candidate in the current study.

## Discussion

In recent years, mRNA technology has provided a new appealing approach for the development of antibody therapies. Notably, several mRNA-encoded antibodies against infectious agents, toxins, and tumors have been developed and have shown superior efficacy compared with that of the classical protein antibody format for disease treatments in preclinical and clinical trials.^30^

Previously, Hooper JW and his colleagues attempted to demonstrate the feasibility of LNP delivery of mRNAs encoding three previously described monoclonal antibodies targeting poxviruses, c7D11, c8A, and c6C, in rabbits via intramuscular injection. However, intramuscular injection of mRNA-LNPs showed limitations in logistical issues and lower circulating levels of effector antibodies in the study by Hooper JW and his colleagues. Based on empirically derived target serum antibody levels that were unlikely to provide protection, they did not perform further challenge testing.^31^ In the current study, utilizing our well-established mRNA antibody platform, we report the first mRNA antibodies that provided protective potency against poxvirus via intravenous administration in the VACV-WR lethal challenge mouse model. Our data showed that the protective capacity of the mRNA antibody is closely related to the neutralization titers. Moreover, besides the influence of in vivo IgG expression differences, the protective capacity of the original mAbs against VACV infection may also be responsible the protection efficacy. The protection efficacy of the original human mAbs in protein format, which targeted the EEV surface proteins VACV A33 and VACV B5 and the IMV surface proteins MPXV M1 and VACV A27, has been well characterized previously. Gilchuk et al. proved that the most efficient protection required the cooperation of antibodies that target different viral proteins at distinct stages of maturation of the virus.^25^ Consistently, our data demonstrate that the double-hit mRNA antibody cocktail Mix2a targeted both EEV-form and IMV-form viruses and exhibited superior protective potency among all the candidates. Due to the limited access to the VACV challenge facilities, the therapeutic efficacy of our mRNA antibodies was not performed, and long-term pharmacokinetics with clinical batch will be warranted in the future.

To combat the current mpox outbreaks, strategies to repurpose and discover novel vaccines are being researched. Several multicomponent mpox mRNA vaccine candidates, including 2-valent, 4-valent, 5-valent, and 6-valent candidates, have been reported.^32–34^ Among these novel mRNA vaccine candidates, the EEV envelope glycoproteins B6 and A35 and the IMV surface membrane proteins M1, A29, E8, and H3 were the main selected antigens. These studies confirmed the findings that the most efficient vaccine candidates require both EEV- and IMV-targeted antigens. As the representative protective proteins of EEV- and IMV-form viruses respectively, MPXV A35 (the homolog of VACV A33) and MPXV M1 are important targets for the development of genetically engineered vaccines and therapeutics. Besides, previous studies have shown that binding antibodies against MPXV A35, M1 proteins and VACV A33 could be detected from smallpox-vaccinated or MPXV infected individuals.^35–38^ Furthermore, several anti-A33 monoclonal antibody and anti-M1 monoclonal antibody have exhibited potent antiviral efficacy in vitro and in animal challenge models.^39,40^ Previously, Hou et al. discovered that mRNA encoding MPXV antigen combination A35 + M1 can provide complete protection for mice with lethal vaccinia virus challenge.^41^ Recently, Wang et al. reported a ‘two-in-one’ immunogen based on the single-chain dimeric MPXV A35 bivalently fused with M1 (termed DAM) drives potent immune response and in vivo protective efficacy.^42^ Similarly, our data show that a combination of A33-targeted+M1-targeted mRNA antibodies mRNA antibodies exhibited superior protection potency.

Notably, two mRNAs encoding the light and heavy chains were encapsulated into LNP formulations. Correct pairing of cognate light and heavy chains is critical for antibody functions, which makes the multicomponent mRNA antibodies slightly more complex than the multicomponent mRNA vaccines.^43,44^ Previous studies have shown that when different antibodies were generated from mRNAs in vitro, compared with applying only one antibody, the levels of functional antibodies were lower when the different antibodies were administered simultaneously. In vivo administering different mRNA antibodies simultaneously from “one syringe” might lead to inefficient production of functional antibodies.^31^ In the current study, we also found that compared with those from applying only one mRNA antibody, the levels of functional antibodies from the Mix2a and Mix2b groups were slightly lower. The slight differences may be attributed to the interference between different mRNA antibodies. As mRNA-encoded antibodies are still in the early stages of development, while the interference between different mRNA antibodies is a possible explanation for the observed differences in functional antibody levels, other factors may also contribute to these differences. For multicomponent mRNA antibodies, the appropriate proportion of different components is a major concern. Adjusting the ratios of different mRNA antibody components to optimize the effects in future experiments is worth considering. Besides, to better understand the potential interferences, further studies to determine the specific mechanisms by which different mRNA antibodies interact and affect each other’s function is needed.

Collectively, our present results clearly demonstrate that a single injection with the mRNA antibody cocktail Mix2a provide protection against orthopoxvirus infection in mice. Human antibodies from tailored mRNA combinations represent a promising antiviral strategy to combat current and future human poxvirus outbreaks.

## Materials and methods

### Ethics statement

Experiments involving live poxviruses were performed in the biosafety level 2 (BSL-2) facilities in the Beijing Institute of Microbiology and Epidemiology, Academy of Military Medical Sciences (AMMS). All animal studies were performed in strict accordance with the guidelines set by the Chinese Regulations of Laboratory Animals and Laboratory Animal-Requirements of Environment and Housing Facilities. All animal experiments were approved by the Experimental Animal Committee of Laboratory Animal Center, AMMS (approval number: IACUC-DWZX-2022-055).

### Cells and viruses

BHK-21 (ATCC, #CCL-10) and 293TN (LV900A-1, System Biosciences) cells were grown in Dulbecco’s modified Eagle’s medium (DMEM) supplemented with 10% FBS and 1% HEPES. BS-C-1 (ATCC, #CCL-26) cells were grown in Minimum Essential Medium Eagle (MEM) contained non-essential amino acids and supplemented with 10% FBS and 1% Pen/Strep (Procell). Vaccinia virus (VACV) WR strain (GenBank: AY243312.1) was obtained from the Institute of Microbiology, Chinese Academy of Sciences. EEV or IMV forms of VACV were prepared as described elsewhere.^45,46^ These viruses were passaged and titrated by standard plaque assay as previously described in BS-C-1 cells.^47^

### mRNA preparation

DNA sequences respectively encoding the human IgK light chain signal peptide and the codon-optimized heavy chain (HC) and light chain (LC) region of four anti-orthopoxvirus monoclonal antibodies (mAbs), termed as mAb22, mAb283, mAb26 and mAb301, were synthesized by Sangon Biotech (Shanghai, China). The final HC and LC mRNAs (mRNA-mab22-HC, mRNA-mab22-LC, mRNA-mab283-HC, mRNA-mab283-LC, mRNA-mab26-HC, mRNA-mab26-LC, mRNA-mab301-HC, and mRNA-mab301-LC) were then produced in vitro using T7 RNA polymerase-mediated transcription from the linearized DNA plasmids.

### Identification of mRNAs in vitro expression

One day before transfection, BHK-21 cells and 293TN cells were seeded in 6-well plates with 10^5^ cells/well and 5 ×10^5^ cells/ well, respectively. Cells were transfected with HC mRNA and LC mRNA (at the molar ratio of 1:1) using Lipofectamine Messenger MAX (Thermo Fisher Scientific) according to the manufacturer’s instructions. At 24 h after transfection, the supernatants were harvested and used for analysis. For western blot analysis, the supernatants were respectively separated on an 8–20% Precast-Glgel Tris-Glycine PAGE (Sangon Biotech) under reduced conditions, and then transferred onto a blotting membrane using HRP-conjugated goat anti-human IgG-Fc (ZSBiO) at 1:5000 and HRP-conjugated goat anti-human F(ab’)_2_ (abcam) at 1:10,000.

### Human IgG ELISA assay

The human IgG levels in the samples were quantified using the Human IgG ELISA Kit (Sangon Biotech) according to the manufacturer’s instructions. Briefly, standards and diluted samples were respectively added to the pre-coated micro well plate, and incubated for 90 min at 37 °C. Then the biotin-conjugated antibody working solution was added and incubated for 60 min at 37 °C. After five times washing, the HRP-conjugated streptavidin working solution was added and incubated for 30 min at 37 °C. After five times washing, substrate reagent was added to the plate. The color was developed at 37 °C for about 15 min, and then the reaction was stopped by the stop solution. The OD value at a wavelength of 450 nm/630 nm was immediately measured using Synergy H1 hybrid multimode microplate reader (BioTek).

### Orthpoxvirus-specific ELISA assay

Orthpoxvirus-specific ELISA analysis was used to identify the binding activity. Briefly, 96-well ELISA plates were respectively coated with the recombinant A35 (Sino Biological), B6 (Atagenix), M1 (Atagenix), A29 (Atagenix) protein of MPXV and heat-inactivated VACV WR strain (GenBank: AY243312.1) and incubated at 4 °C overnight. The plate was washed five times and blocked with PBS containing 5% nonfat milk powder for 2 h. Then, the plate was washed five times and samples were added to the plates and incubated at 37 °C for 2 h. The plate was washed five times and then HRP-conjugated goat anti-human IgG-Fc (1:5000, ZSBiO) was added to the plate. After 1 h of incubation at 37 °C, the plate was washed five times. Then the Soluble TMB (Cwbio) was added and incubated for 15 min at room temperature. The reaction was stopped by stop solution (Solarbio) and the absorbance at 450 nm/630 nm was immediately measured by Synergy H1 hybrid multimode microplate reader (BioTek). For serum samples, the IgG endpoint titers were defined as the highest reciprocal serum dilution that yielded an absorbance >2-fold over background values.

### LNP encapsulation of mRNA

LNP formulation was conducted using the established procedure as described previously.^29^ Briefly, the mRNAs encoding mAbs were mixed at a molar ratio of 1:1 (HC mRNA over LC mRNA) for all antibodies. The lipid mixtures containing ionizable lipids, 1-,2-distearoyl-sn-glycero-3-phosphocholine (DSPC), cholesterol and PEG-lipid (with molar ratios of 50:10:38.5:1.5) were combined with mRNA mixtures using the NanoAssemblr® Ignite^+^ mixer (Precision Nanosystems). The formulation was diafiltrated with 10× volume of PBS through a tangential flow filtration membrane (100 kD), and filtrated using a 0.22 μm filter. The final mRNA-LNP formulations (termed as mRNA-mab22-LNP, mRNA-mab283-LNP, mRNA-mab26-LNP and mRNA-mab301-LNP) were stored at 2–8 °C until use, and the particle size and distribution were measured using dynamic light-scattering assay as described previously.^48^ The mRNA antibody cocktail candidates Mix2a and Mix2b were developed by mixing mRNA-mab22-LNP and mRNA-mab26-LNP, mRNA-mab283-LNP and mRNA-mab26-LNP, respectively. Empty LNPs were utilized as the placebo.

### Animal injection experiments

Six- to eight-week-old specific pathogen-free female BALB/c mice were purchased from Vital River Laboratory Animal Technology Co., Ltd. For the identification of in vivo expression, BALB/c mice (*n* = 6 per group) were respectively injected intravenously (i.v.) with a single dose of 1 mg/kg of mRNA-mab22-LNP, mRNA-mab283-LNP, mRNA-mab26-LNP, mRNA-mab301-LNP and Placebo, with the injection volume of 50 μL. Orbital blood from each group of mice were collected at 24 h post injection. For the evaluation of mRNA antibody cocktail candidates Mix2a and Mix2b, BALB/c mice (*n* = 6 per group) were respectively injected intravenously (i.v.) with 2 mg/kg of Mix2a (1 mg/kg mRNA-mab22-LNP plus 1 mg/kg mRNA-mab26-LNP), Mix2b (1 mg/kg mRNA-mab22-LNP plus 1 mg/kg mRNA-mab301-LNP) and Placebo, with the injection volume of 100 μL. At days 1, 3, and, 7 post injection, orbital blood was collected. Blood samples were centrifuged at 3000 rpm at 4 °C for 10 min, and then sera were harvested and heat inactivated for 30 min at 56 °C before a further test.

### Virus neutralization assays

The neutralizing antibody titers were measured using EEV or IMV forms of VACV by plaque reduction neutralization (PRNT) assay, and presented as 50% plaque reduction neutralization titer (PRNT_50_). The PRNT test was performed in BS-C-1 cells. Neutralization of VACV IMV particles was performed in the presence of 10% guinea pig complement (Solarbio). Neutralization of VACV EEV particles was performed in the presence of 10% baby rabbit complement (Solarbio) and 20 μg/mL anti-M1R mAb (Atagenix). Cells with 0.5% methylcellulose in DMEM with 2% inactivated FBS overlay were incubated at 37 °C in 5% CO_2_ for 2 days, and then fixed and stained with crystal violet for the plaque visualization. The PRNT_50_ values were calculated by the Spearman-Karber method.

### Protection against VACV challenge in mice

Six- to eight-week-old specific pathogen-free female BALB/c mice (*n* = 5-6 per group) were respectively injected intravenously (i.v.) with a single dose of mRNA-mab22-LNP, mRNA-mab283-LNP, mRNA-mab26-LNP, mRNA-mab301-LNP, Mix2a, Mix2b and placebo. In BSL-2 facilities, all mice were intranasally challenged with 7.5 × 10^4^ PFU VACV (WR strain) at 24 h post administration. Body weight and survival were monitored daily for 7 days. Mice of each group were euthanized and lung, nasal turbinate and liver were harvested at 7 d.p.i.

### Viral genome quantification

Viral nucleic acids in tissues were extracted by using the Viral RNA/DNA Extraction Kits (TIANLONG) and then measured using Probe qPCR Mix (Takara) as previously described.^49^ The primers and probe used for qPCR were VACV-F (5’- GGCAATGGATTCAGGGATATAC -3’), VACV-R (5’- ATTTATGAATAATCCGCCAGTTAC -3’), and VACV-P (5’- FAM- CAATGTGTCCGCTGTTTCCGTTAATAAT -BHQ1-3’). Viral genome copies in nasal turbinate, liver and lung were expressed as genomes/g tissue.

### Lung virus titers measurement

Partial of lung tissues were homogenized using a tissue homogenizer (Scientz). Lung homogenates were clarified by centrifugation at 8000 rpm, 10 min at 4 °C. Infectious VACV particles in the supernatants of lung homogenates were determined by standard plaque assay in BS-C-1 cells as described previously.^49^ Lung virus titers were expressed as PFU/g of tissue.

### Histology and immunohistochemistry analysis

Partial of lung tissues from mice were fixed with 4% formaldehyde (biosharp) for over 48 h and embedded in paraffin according to standard histological assays. Sections of the paraffin-embedded tissues 3 μm thick were cut and placed on glass histology slides. The slides were further stained with hematoxylin and eosin (H&E). For immunohistochemistry (IHC) analysis, anti-VACV D8 mAb (mAb249) were used. The slides were scanned with a Pannoramic MIDI histoscanner (3DHISTECH) and images were analyzed using Pannoramic Viewer software.

### Statistical analysis

Statistical analyses were carried out using GraphPad Prism version 9.0.0 (GraphPad software). Data are shown as mean ± SD. Significance was calculated using One-way ANOVA (*<0.05, **<0.01, ***<0.001 and ****<0.0001).

### Supplementary information

Supporting Material

## Data Availability

The data that support the findings of this study are available from the corresponding author upon reasonable request.
