# Supplementary material for: Rapid development of double-hit mRNA antibody cocktail against orthopoxviruses
Source: Signal Transduct Target Ther. 2024 Mar 27;9:69. doi: 10.1038/s41392-024-01766-8 (PMC10966106; doi:10.1038/s41392-024-01766-8)
Supplement: Supplementary file 1 — Supporting Material [file 41392_2024_1766_MOESM1_ESM.docx]

Supplementary Materials for

Rapid Development of Double-hit mRNA Antibody Cocktail against Orthopoxviruses

Hang Chi^1^, Suo-Qun Zhao^1^, Ru-Yi Chen^1^, Xing-Xing Suo^1,2^, Rong-Rong Zhang^1^, Wen-Hui Yang^1^, Dong-Sheng Zhou^1^, Min Fang^3^, Bo Ying^4^, Yong-Qiang Deng^1, *^, Cheng-Feng Qin^1,5, *^

Correspondence to: Yong-Qiang Deng (dengyq1977@126.com) & Cheng-Feng Qin (qincf@bmi.ac.cn)

**This PDF file includes:**

Supplementary figures 1-2

Supplementary tables 1-2


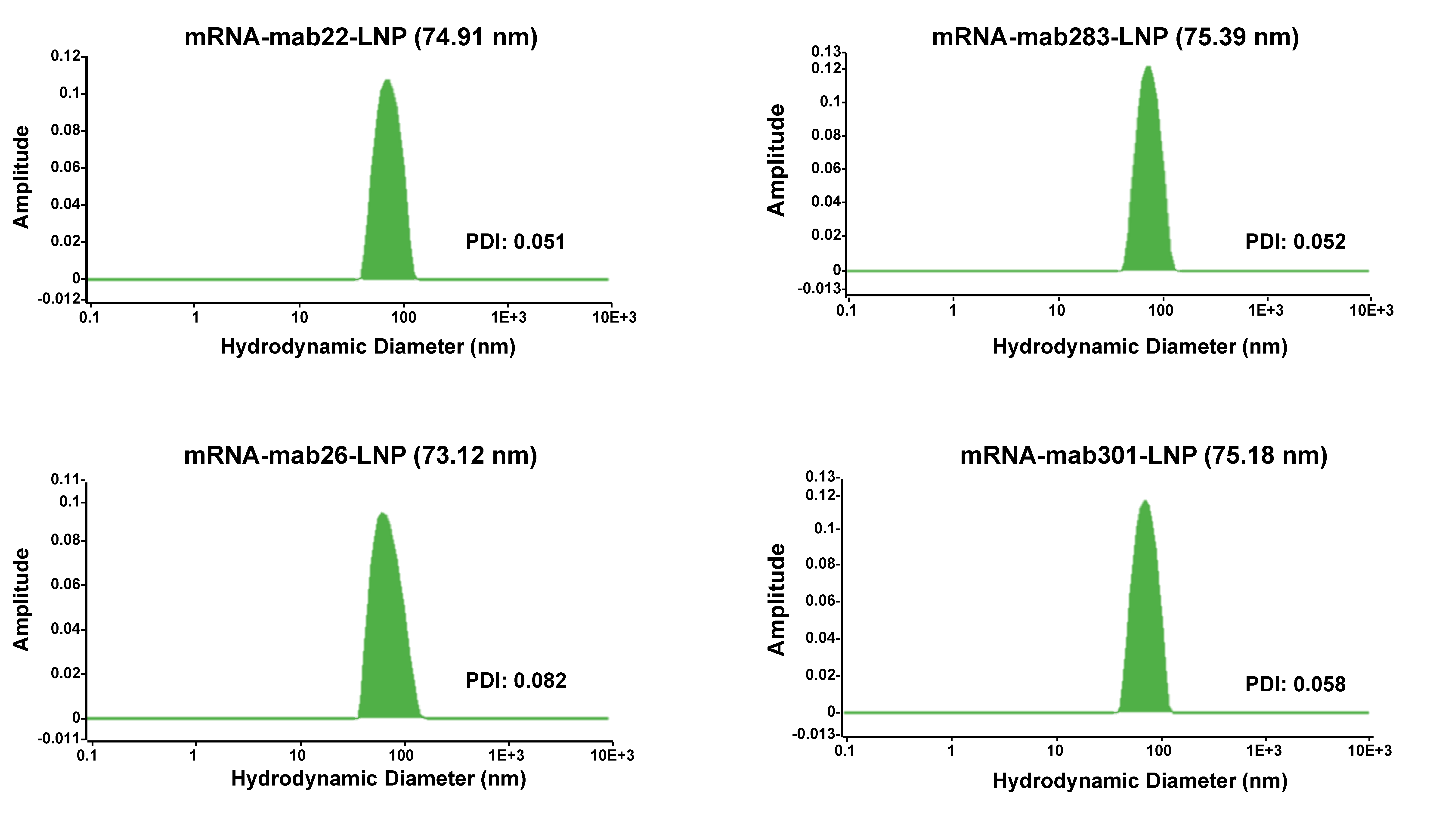


Supplementary Figure 1. The particle size of four LNP-encapsulated mRNA encoding antibodies.

The particle size and distribution of mRNA-mab22-LNP, mRNA-mab283-LNP, mRNA-mab26-LNP and mRNA-mab301-LNP.


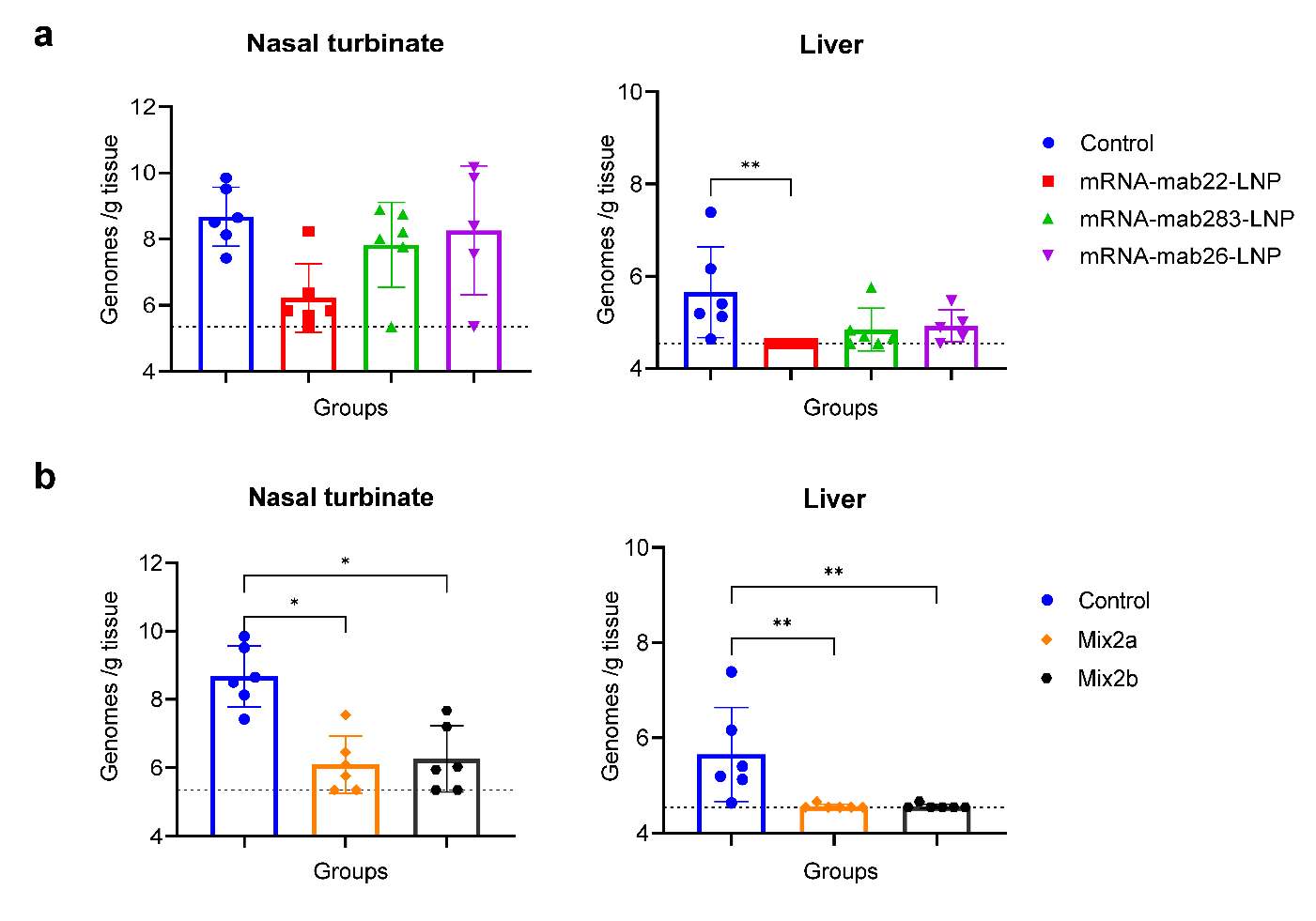


Supplementary Figure 2. Viral genome copies in nasal turbinate and liver at 7 d.p.i.

Viral genome copies in lungs of mice received **(a)** the candidate mRNA antibody component and **(b)** mRNA antibody cocktail.

Supplementary Table 1. The homology of the four antigens among VACV, VARV, MPXV and CPVX.

| **Antigens** | **MPXV** | **VARV** | **VACV** | **CPVX** |
| --- | --- | --- | --- | --- |
| EEV envelope glycoprotein | A35 | A36 | A33 | A34 |
|  | B6 | B7 | B5 | B4 |
| IMV surface membrane protein | M1 | M1 | L1 | L1 |
|  | A29 | A30 | A27 | A28 |

Supplementary Table 2. The sequences of the mRNA constructs.

| **Name** | **Sequences** | **Length（nt）** |
| --- | --- | --- |
| **mab22-LC** | GCCAUUGUGAUGACACAAAGCCCUGCUACUCUCAGCGUGUCCCCCGGCGAGAGGGCCACACUGAGCUGCCGGGCCAGCCAGUCCGUGAGCAGCACACUGGCCUGGUACCAGCAGAAGCCCGGCCAGGCCCCCCGGCUGCUGAUCUACGGGGCCUCCACAAAGGCCACCGGCAUUCCCGCCCGGUUCAGCGGGAGCGGGAGCGGGACAGAGUUCACCCUGACCAUCAGCAGCCUGCAGUCUGAGGACUUCGCCGUGUACUACUGCCAGCACUACAACAACUGGCCCCCCCUGCUGACCUUCGGGGGCGGCACCAAGGUGGAUAUCAAGACAGUGGCCGCCCCCUCCGUGUUCAUCUUCCCCCCCAGUGAUGAGCAGCUGAAGUCCGGCACAGCCAGCGUGGUGUGCCUGCUGAACAACUUCUACCCCAGGGAGGCCAAGGUGCAGUGGAAGGUCGACAACGCCCUGCAGAGCGGGAACAGCCAGGAGAGCGUGACCGAGCAGGAUAGCAAGGAUAGCACAUACAGCCUGUCCUCCACCCUGACACUGAGCAAGGCCGACUACGAGAAGCACAAGGUGUACGCCUGCGAGGUGACACACCAGGGGCUGAGCAGCCCCGUGACAAAGAGCUUCAACAGGGGCGAGUGC | 645 |
| **mab22-HC** | CAAGUGCAGCUGGUGGAGAGCGGCGGCGGCGUGGUGCAGCCCGGCCGGAGCCUGCGGCUCAGCUGCGCCGCCAGCGGGUUCACCUUCAGCAACAGCGGGAUGCACUGGGUCCGGCAGGCCCCCGGCAAGGGGCUGGAGUGGGUGGCCGUGAUCUGGUUCGACGGCACCAACAAGUACUACGCCGACAGCGUGAAGGGCAGGUUCACAAUCAGCAGGGAUAACUCCAAGAACACCCUGUACCUGCAGAUGAACAGCCUGCGGGCCGAGGACACAGCCGUGUACUACUGCGCCAGGGUGCCCUGCGGGGGCGAUUGCUACUCCGGCUACCUGCAGCACUGGGGCCAGGGCACACUCGUGACCGUGUCCUCCGCAUCCACAAAGGGCCCCUCCGUGUUUCCCCUGGCCCCCAGCAGCAAGAGCACAAGCGGGGGGACCGCCGCCCUCGGGUGCCUGGUCAAGGAUUACUUCCCCGAGCCCGUGACCGUGUCUUGGAACUCCGGCGCCCUGACCUCCGGGGUCCACACCUUCCCCGCCGUCCUGCAGAGCUCCGGGCUGUACAGCCUGAGCAGCGUGGUGACAGUGCCCAGCUCCAGCCUGGGGACCCAGACAUACAUUUGCAACGUGAACCACAAGCCCUCAAACACCAAGGUGGAUAAGCGGGUGGAGCCCAAGAGCUGCGACAAGACCCACACAUGCCCCCCCUGCCCUGCCCCCGAGGCCGCCGGGGGCCCUUCAGUGUUCCUGUUCCCCCCCAAGCCUAAGGAUACACUGUACAUUACCAGGGAGCCCGAGGUCACAUGCGUGGUGGUGGACGUGAGCCACGAGGACCCCGAGGUGAAGUUCAACUGGUACGUCGAUGGGGUGGAGGUGCACAACGCCAAGACAAAGCCCAGGGAGGAGCAGUACAACAGCACCUACCGGGUGGUGAGCGUGCUGACAGUGCUGCACCAGGACUGGCUGAACGGGAAGGAGUACAAGUGCAAGGUGUCCAACAAGGCCCUGCCAGCCCCCAUUGAAAAGACCAUCUCUAAGGCCAAGGGGCAGCCCAGGGAGCCCCAGGUGUACACACUGCCACCCAGCCGGGAGGAGAUGACCAAGAACCAGGUGAGCCUGACCUGCCUGGUGAAGGGGUUCUACCCCAGCGAUAUCGCCGUGGAGUGGGAGAGCAAUGGCCAGCCCGAGAACAACUACAAGACAACACCCCCUGUGCUGGAUAGCGACGGGAGCUUCUUCCUGUACUCCAAGCUGACAGUGGAUAAGUCCAGGUGGCAGCAGGGGAACGUGUUCAGCUGCUCCGUGAUGCACGAGGCCCUGCACAACCACUACACCCAGAAGUCCCUGAGCCUGUCUCCCGGCAAG | 1359 |
| **mab283-LC** | GAUAUCGUGAUGACACAGAGCCCCCUGAGCCUGCCCGUGACACCCGGCGAGCCCGCCAGCAUCAGCUGCAGGAGCUCCCAGAGCCUGCUGCACAGCAACGGCUACAACUACCUGGACUGGUACCUGCAGAAGCCCGGCCAGAGCCCCCAGCUGCUGAUCUACCUGGGCUCCAACCGGGCCAGCGGCGUGCCCGACCGGUUCAGCGGGUCCGGCAGCGGGACAGACUUCACCCUGAAGAUUAGCAGGGUCGAGGCCGAGGAUGUGGGGGUGUACUACUGUCUCCAGGCCCUGCAGACCCUGCCCAUUACCUUCGGGCAGGGCACCAGGCUGGAGAUCACCGUGGCCGCUCCCAGCGUGUUCAUCUUCCCCCCCAGCGAUGAGCAGCUGAAGUCCGGCACAGCCAGCGUGGUGUGCCUGCUGAACAACUUCUACCCCAGGGAGGCCAAGGUGCAGUGGAAGGUGGAUAACGCCCUGCAGUCCGGGAAUAGCCAGGAGUCCGUGACCGAGCAGGACAGCAAGGACAGCACCUACUCCCUGAGCAGCACCCUGACACUGUCCAAGGCCGACUACGAGAAGCACAAGGUGUACGCCUGCGAGGUGACCCACCAGGGGCUGAGCUCCCCCGUGACAAAGAGCUUCAACCGGGGCGAGUGC | 654 |
| **mab283-HC** | CAAGTGCAGCTGGTCCAGAGCGGCGCCGAGGTGAAGAAGCCCGGCAGCAGCGTGAAGGTGAGCTGCAAGGCCAGCGGGGGCACATTTTCCACCTACGCCATCAACTGGGTGCGGCAGGCCCCCGGCCAGGGGCTGGAGTGGATGGGGAGGATCATTCCCATCCTGGGGACCGCCAACTACGCCCAGAAGTTTCAGGGCCGCGTCACAATCACCGCCGATAAGAGCACAAGCACCGCCTACATGGAGCTGTCCTCCCTGCGGAGCGAGGATACCGCCGTGTACTACTGCGCCAGGCGGGGCGGGGAGGGCGCCGCCCACGGGATGGACGTCTGGGGGCAGGGCACCACCGTGACCGTGAGCTCCGCCAGCACCAAGGGCCCCAGCGTCTTTCCCCTGGCCCCCAGCTCCAAGAGCACCAGCGGGGGGACAGCCGCCCTGGGGTGCCTGGTGAAGGACTACTTCCCCGAGCCAGTGACAGTGTCCTGGAACAGCGGGGCCCTGACCAGCGGGGTGCACACATTCCCCGCCGTCCTGCAGAGCAGCGGCCTCTACAGCCTGAGCAGCGTGGTGACAGTGCCTAGCTCCAGCCTGGGCACCCAGACCTACATCTGCAACGTGAACCACAAGCCTAGCAACACCAAGGTGGATAAGAGGGTGGAGCCTAAGAGCTGCGATAAGACACACACCTGCCCCCCCTGCCCCGCCCCTGAGGCCGCCGGGGGCCCCAGCGTGTTCCTGTTCCCCCCCAAGCCCAAGGACACCCTGTACATCACACGGGAGCCTGAGGTCACCTGCGTCGTGGTGGACGTGAGCCACGAAGATCCCGAGGTGAAGTTTAACTGGTACGTGGACGGCGTGGAGGTGCACAACGCCAAGACAAAGCCACGGGAGGAGCAGTACAACTCCACATATCGGGTCGTGTCCGTGCTGACAGTGCTGCACCAGGATTGGCTCAACGGCAAGGAGTACAAGTGCAAGGTGAGCAACAAGGCCCTGCCCGCCCCTATCGAAAAGACCATTAGCAAGGCCAAGGGCCAGCCCAGGGAGCCCCAGGTGTACACACTGCCCCCCAGCAGGGAGGAGATGACAAAGAACCAGGTGAGCCTGACCTGCCTCGTGAAGGGGTTCTACCCCTCCGATATTGCCGTGGAGTGGGAGTCCAACGGCCAGCCCGAGAACAACTACAAGACCACCCCCCCCGTGCTGGACAGCGACGGCAGCTTCTTCCTGTACAGCAAGCTGACCGTGGATAAGAGCAGGTGGCAGCAGGGCAACGTCTTTAGCTGCTCCGTGATGCACGAGGCCCTGCACAACCACTATACACAGAAGTCCCTTAGCCTCTCCCCCGGCAAG | 1353 |
| **mab26-LC** | CAGUCCGCCCUGACCCAGCCCCCCAGCGCCAGCGGCAGCCCCGGCCAGAGCGUGACCAUCACCUGCACAGGGUCCAGCAGCGACGUGGGGGGCUACAACUACGUGAGCUGGUACCAGCAGCACCCCGGCAAGGCCCCUAAGGUCGUGAUCUACGAGGUGAACAAGCGGCCCUCCGGGGUCCCCCACCGGUUCUCCGGGAGCAAGUCCGGGAACACCGCCAGCCUGACAGUGAGCGGGCUCCAGGCCGAGGAUGAGGCCGACUACUACUGCUCCAGCUACGCCGGCACCGAGACAGUGGCCUUCGGCGGGGGCACUAAGCUGACCGUGCUGGGCCAGCCUAAGGCCGCCCCCUCCGUGACACUGUUCCCACCCUCCAGCGAGGAGCUGCAGGCCAACAAGGCCACCCUGGUGUGCCUGAUCUCCGACUUCUACCCUGGGGCCGUGACCGUGGCCUGGAAGGCCGAUAGCAGCCCCGUGAAGGCCGGCGUGGAGACAACAACACCCAGCAAGCAGAGCAACAACAAGUACGCCGCCAGCAGCUACCUGUCCCUGACCCCCGAGCAGUGGAAGUCCCACAGGAGCUACAGCUGCCAGGUCACCCACGAGGGCAGCACAGUGGAAAAGACAGUGGCCCCCACAGAGUGCAGC | 648 |
| **mab26-HC** | CAGGTCCAGCTGGTCCAAAGCGGCGGCGGGCTCATTCAGCCCGGCGGGTCCCTGCGGCTCTCTTGTGTCGTGAGCGGCTTCAACGTGGCCACCAACTACATGAGCTGGGTGAGGCAGGCCCCCGGCAAGGGGCTGGAGTGGGTGAGCGTGATCTACAGCGGCGGCTCCACCTACTACGCCGACTCCGTGAAGGGGAGGTTTACTATTAGCCGGGACAACAGCAAGAACACAGTGTTCCTGCAGATGAACTCCCTGCGGCCCGAGGATACAGCCGCCTACTACTGCGCCAAGGGCGGGGGCCTGGGGCTGGATTACTGGGGGCAGGGCACCCTCGTGACCGTGAGCAGCGCCAGCACCAAGGGCCCCTCCGTGTTCCCCCTGGCCCCCAGCAGCAAGTCCACCAGCGGCGGGACAGCCGCCCTGGGGTGCCTGGTGAAGGATTACTTTCCCGAGCCAGTGACAGTGAGCTGGAACTCCGGGGCCCTGACAAGCGGCGTGCACACATTCCCCGCCGTGCTCCAGTCCTCCGGGCTCTACAGCCTGTCCTCCGTCGTGACCGTGCCATCCAGCAGCCTGGGGACACAGACCTACATCTGCAACGTGAACCACAAGCCCAGCAACACAAAGGTGGATAAGCGGGTGGAGCCCAAGTCCTGCGATAAGACACACACATGCCCCCCCTGCCCCGCCCCCGAGGCCGCCGGCGGGCCTAGCGTGTTTCTGTTCCCCCCCAAGCCCAAGGACACCCTGTACATCACCAGGGAGCCCGAGGTGACCTGCGTGGTGGTGGATGTGAGCCACGAAGATCCTGAGGTGAAGTTTAACTGGTACGTGGACGGGGTGGAGGTGCACAACGCCAAGACCAAGCCCAGGGAGGAGCAGTACAACAGCACATACCGGGTGGTGAGCGTGCTGACCGTGCTGCACCAGGACTGGCTGAACGGGAAGGAGTACAAGTGCAAGGTGAGCAACAAGGCCCTCCCCGCCCCCATCGAGAAAACCATCTCCAAGGCCAAGGGGCAGCCACGGGAGCCCCAGGTGTACACCCTGCCCCCAAGCAGGGAGGAGATGACAAAGAACCAGGTGTCCCTGACATGCCTGGTGAAGGGGTTTTACCCCTCCGACATCGCCGTGGAGTGGGAGAGCAACGGGCAGCCCGAGAACAACTACAAGACCACCCCCCCTGTGCTGGACAGCGACGGGAGCTTCTTCCTGTACTCCAAGCTGACAGTCGATAAGAGCCGGTGGCAGCAGGGCAACGTGTTCAGCTGCAGCGTGATGCACGAGGCCCTGCACAACCACTACACACAGAAGTCCCTGAGCCTCAGCCCAGGGAAG | 1338 |
| **Mab301-LC** | UCCUACGAGCUGACACAGAGCCCCAGCGUGUCCGUGAGCCCCGGCCAGACUGCCAGGAUCACAUGCAGCGGCGACGCCCUGCCCGAGCAGUACGCCUACUGGUACCAGCAGAAGCCCGGCCAGGCCCCCGUGCUCGUGAUCUACAAGGACAGCGAGCGGCCCUCCGGCAUUCCCGAGCGGUUCAGCGGCAGCGGCAGCGGUACAACAGUGACCCUGACCAUCACAGGGGUGCAGGCCGAGGAUGAGGCCGAUUACUACUGCCAGAGCGCCGAUAACAGCGGGACAUACGAGGUGUUCGGGACCGGGACCAAGGUGACUGUGCUGGGGCAGCCCAAGGCCGCCCCCAGCGUGACACUGUUCCCCCCCUCCAGCGAGGAGCUGCAGGCCAACAAGGCCACCUUAGUGUGUCUGAUCUCAGACUUUUACCCUGGCGCCGUCACAGUGGCCUGGAAGGCCGACAGCAGCCCCGUCAAAGCUGGCGUGGAAACCACAACCCCCUCCAAACAGUCCAACAACAAAUACGCCGCCAGCUCCUACCUGAGCCUGACACCCGAGCAGUGGAAGUCCCACCGGAGCUACAGCUGCCAGGUGACACACGAGGGGAGCACCGUGGAAAAGACAGUGGCCCCCACCGAGUGCAGC | 642 |
| **Mab301-HC** | GAAGUGCAGCUGCUGGAGUCCGGCGGCGGCCUGGUGCAGCCCGGCGGCAGCCUGCGGCUGAGCUGCGCCGCUAGCGGGUUCAGCUUCUCCAGCUACGCCAUGAGCUGGGUGCGGCAGGCCCCCGGCAAGGGCCUGGAGUGGGUGAGCGGGAUCGGGAACUCCGGGGAUCGGACCUUCUACGCCGAUAGCGCCAAGGGGAGGUUCACCAUCUUCAGAGACAACAGCAACAACAGGCUGUACCUGCAGAUGAACUCCCUGCGGGCCGCCGACACCGCCGUCUACUACUGCGCUAAGUGGGGCCGGUUUGAGUCCGGGGCCUUCUGGGGGCAGGGCGUGCUGGUGACCGUGAGCUCCGCCAGCACCAAGGGCCCAAGCGUGUUCCCACUGGCCCCUAGCAGCAAGAGCACUAGCGGGGGCACCGCCGCCCUGGGGUGCCUGGUGAAGGAUUACUUCCCCGAGCCCGUGACAGUGAGCUGGAACUCCGGGGCCCUCACAUCCGGCGUGCACACAUUCCCCGCCGUGCUGCAGUCCAGCGGGCUCUACAGCCUCAGCAGCGUGGUGACAGUGCCCUCCUCCAGCCUCGGGACCCAGACAUACAUCUGCAACGUGAACCACAAGCCCAGCAACACAAAGGUGGAUAAGCGCGUGGAGCCCAAGAGCUGCGACAAGACACACACAUGCCCCCCCUGCCCCGCCCCUGAGGCCGCCGGCGGGCCCAGCGUGUUUCUGUUCCCCCCUAAGCCCAAGGAUACACUGUACAUUACAAGGGAGCCCGAGGUCACUUGCGUGGUGGUGGAUGUGAGCCACGAGGACCCUGAGGUGAAGUUCAACUGGUACGUCGAUGGGGUGGAGGUGCACAACGCCAAGACCAAGCCCAGGGAGGAGCAGUACAACAGCACCUACAGGGUGGUGUCCGUGCUGACAGUGCUGCACCAGGACUGGCUGAACGGGAAGGAGUACAAGUGCAAGGUGAGCAACAAGGCCCUCCCAGCCCCCAUUGAAAAGACCAUCAGCAAGGCCAAGGGCCAGCCCAGGGAGCCCCAGGUGUACACACUCCCCCCCAGCCGGGAGGAGAUGACAAAGAACCAGGUGAGCCUGACCUGCCUGGUGAAGGGGUUCUACCCCUCCGACAUUGCCGUGGAGUGGGAGUCCAACGGGCAGCCCGAGAACAACUACAAGACCACCCCCCCCGUGCUGGACAGCGACGGGAGCUUCUUCCUGUACAGCAAGCUGACUGUCGAUAAGAGCAGGUGGCAGCAGGGGAACGUGUUCAGCUGCUCCGUGAUGCACGAGGCCCUGCACAACCACUACACCCAGAAGUCCCUGAGCCUGAGCCCCGGCAAG | 1344 |
| **5’-UTR** | CUUGUUCUUUUUGCAGAAGCUCAGAAUAAACGCUCAACUUUGGC | 44 |
| **Signal Peptide** | AUGGAGCUGGGACUGAGCUGGAUUUUUCUGCUGGCCAUUCUGAAGGGGGUGCAGUGC | 57 |
| **3’-UTR** | UAGGCUGGAGCCUCGGUGGCCAUGCUUCUUGCCCCUUGGGCCUCCCCCCAGCCCCUCCUCCCCUUCCUGCACCCGUACCCCCGUGGUCUUUGAAUAAAGUCUGAGUGGGCGGCA | 114 |
